# Progress in Surgery

**Published:** 1903-12-05

**Authors:** 


### Progress in Surgery.

### HERNIA.

In an interesting paper Edward Deanesley [1] assails the commonly accepted ideas on the causation of hernia. He states that almost all oblique inguinal herniæ are of congenital origin. In support of this statement he adduces the following arguments :—

1. Many oblique inguinal herniæ are undoubtedly congenital, as proved by the descent of gut into the tunica vaginalis.

2. In traumatic herniæ, such as those which occur in scars caused by gradual protrusion of a peritoneal sac, the tumour is spherical and no constriction exists at the neck, whereas in oblique inguinal herniæ the tumour is pear-shaped and a well-marked constriction is always present at the neck.

3. Many large herniæ appear suddenly which shows that a sac must have been there ready to receive the protrusion, and the probability of a sac with a constricted neck being thus suddenly formed is very small.

4. The admittedly congenital herniæ may not make their appearance for years after birth, which shows that the sac has been present all that time, but empty ; it is not, therefore, difficult to believe that many such sacs may exist and never give rise to a hernial protrusion.

5. It has, moreover, often been observed that such a patent process has existed, and yet a hernia into it has not occurred. Sachs stated that he found the process patent in 59 per cent. of male children under four years of age. Obviously a much larger proportion than had herniæ.

6. If the ordinary oblique hernia came down sometimes into a congenital sac, sometimes as a new protrusion, we would expect to find that most herniæ in children were of the undoubtedly congenital type, but this is not the case, the great majority in appearance conform to the ordinary acquired type.

7. The only real proof of the sac of an oblique hernia being acquired would be the finding of such a hernial sac existing together with an unobliterated funicular process ; this has never been found. The writer points out that the question is not one merely of academic interest, but of practical import in the matter of treatment. For he contends that hernia is a congenital defect and one to be remedied like other congenital defects by operation, and this at an early age before the increase in size of the hernia has so stretched and altered the relations of the inguinal canal as to make the cure by operative treatment a much more difficult matter.

From an analysis of the University College Hospital reports relating to cases of strangulated hernia treated over a long period, A. E. Barker [2] arrives at the conclusion that except in very recent cases and among aged patients in a state of great weakness taxis should not be tried. The mortality among cases reduced without operation was over 13 per cent. in the series of cases analysed. The great uncertainty as to the condition of the bowel in a strangulated hernia is a great argument against returning it to the abdomen without inspection, and this argument is supported by the statistics. Again, the acknowledged safety of the operation and possibility of performing a radical cure are weighty considerations in favour of operation.

From a study of cases of gangrenous hernia under his own care [3] he strongly advocates the performance of a primary enterectomy instead of forming an artificial anus. Patients rarely recover after the latter treatment and the results of extensive enterectomy in his hands show a greatly lessened mortality. His reasons in advocating this method are as follows :—The necrosed gut and the fluid in the sac are both septic in the highest degree. The dead bowel and sac must therefore be both removed, for, if left, the patient, already in a state of considerable toxæmia, is in great risk of septicæmia and this in the cases recorded is the usual cause of death.

Then again for a considerable distance above the constriction the bowel is paralysed and distended with most virulent material, which from the condition of the bowel can and does most easily infect the peritoneum, the bowel no longer offering resistance to the passage through its walls of infecting organisms. Even after the establishment of an artificial anus it takes a considerable time for the paralysed bowel to empty itself. The patient's greatest danger is from the continued absorption from the paralysed and toxic bowel. To obviate this danger a considerable length of bowel must be removed, until a fairly healthy portion is reached. The general nutrition seems to suffer but little from the loss of several feet of gut, and the operation of resecting six feet produces no more shock than removing six inches, and takes but little longer to perform.

He describes a simple and time-saving method of dealing with the extra length of mesentery to be divided, folding it over, dividing and tying off two thicknesses at once by means of a Doyen's clamp.

In one case he removed 5 feet 6 inches of distended gut from an old woman 76 years of age. A year afterwards she was in the best of health. He has operated on seven cases of gangrenous hernia in this way with five recoveries, a lower mortality than has been attained in similar cases treated by the establishment of an artificial anus.

Thorburn,[5] in a series of 110 cases of strangulated hernia, had eight cases of gangrenous gut in which he opened the bowel, leaving an artificial anus. Seven of these cases died. In two he resected a few inches ; both of these died within a few hours, so that they were probably past relief from any method of treatment.

These cases bear out Barker's statement that the result is nearly always fatal after the formation of an artificial anus in distended and paralysed gut.

Richter's hernia is a condition which is not merely interesting as a pathological curiosity, but has a very practical importance, for it is a condition which often presents great difficulty in diagnosis and yet has a very high mortality, according to Treves over 60 per cent. The symptoms are aften mild, obstruction not complete, distension slight or appearing late, and frequently no external evidence of hernia. It is most frequently found in femoral or obturator hernia. In two cases recently under the care of Sir W. Collins,[4] the condition occurred in old-standing herniæ, and in both cases the signs of strangulation were recognised in the hernia, they were relieved by herniotomy. J. W. Fraser [6] records a case in which the signs were by no means so clear.

The patient, a woman aged 44, had signs of subacute obstruction, lasting nine days, no sign of hernia was discoverable. The abdomen was opened and a small portion of the circumference of bowel about the size of a hazel nut was found herniated into the femoral opening.

The presence of the bladder in a hernial sac is a condition which has to be borne in mind and recognised by the surgeon. Several cases are on record in which it has been found and not recognised, and consequences more or less serious may result. G. B. Ferguson [7] records a case in his own practice in which the condition was mistaken for a hydrocele of the cord and punctured, it was however sewn up and the patient did well. He refers to a case in the practice of another surgeon in which the protrusion not being diagnosed was ligatured and removed; a few days later urine began to leak from the wound. The diagnosis can generally be settled by the passage of a sound as in a case under the care of Bilton Pollard recorded by H. J. Curtis.[8] The protrusion appeared as a cyst in the posterior wall of the sac, a sound was passed into it from the bladder. It was separated from the hernial sac and pushed back into the abdomen. H. J. Taylor [9] came across the bladder adherent to the neck of right congenital inguinal hernia in a child, the viscus was separated and the sac treated in the usual way. Priestly Leech [10] describes a case of retroperitoneal hernia into the duodenal fossa in a man aged 26. The abdomen was opened for intestinal obstruction. The obstruction was caused by a volvulus of the bowel in the sac, there was no constriction at the neck.

[1] Brit. Med. Jour, June 27, 1903. [2] Lancet, May 30, 1903. [3] Lancet, June 6, 1903. [4] Lancet, May 23, 1903. [5] Brit. Med. Jour., April 25, 1903. [6] Lancet, July 11, 1903. [7] Brit. Med. Jour., July 25, 1903. [8] Brit. Med. Jour., July 11, 1903. [9] Lancet, June 6, 1903. [10] Lancet, June 6, 1903.

